# What is the optimal rate of caesarean section at population level? A systematic review of ecologic studies

**DOI:** 10.1186/s12978-015-0043-6

**Published:** 2015-06-21

**Authors:** Ana Pilar Betran, Maria Regina Torloni, Jun Zhang, Jiangfeng Ye, Rafael Mikolajczyk, Catherine Deneux-Tharaux, Olufemi Taiwo Oladapo, João Paulo Souza, Özge Tunçalp, Joshua Peter Vogel, Ahmet Metin Gülmezoglu

**Affiliations:** UNDP, UNFPA, UNICEF, WHO, World Bank Special Programme of Research, Development and Research Training in Human Reproduction, Department of Reproductive Health and Research, World Health Organization, Avenue Appia 20, Geneva, CH-1211 Switzerland; Brazilian Cochrane Center and Department of Obstetrics, São Paulo School of Medicine, São Paulo Federal University, São Paulo, Brazil; Ministry of Education–Shanghai Key Laboratory of Children’s Environmental Health, Xinhua Hospital, Shanghai Jiao Tong University School of Medicine, Shanghai, China; Epidemiological and Statistical Methods Research Group, Helmholtz Centre for Infection Research, Braunschweig, Germany and Hannover Medical School, Hannover, Germany; INSERM U1153, Obstetrical, Perinatal and Pediatric Epidemiology Research Team, Center for Epidemiology and Statistics Sorbonne Paris Cité, Paris Descartes University, Paris, France; Department of Social Medicine, Ribeirão Preto Medical School, University of São Paulo, Ribeirão Preto, SP Brazil

**Keywords:** Caesarean section, Rates, Population, Maternal mortality, Newborn mortality, Systematic review

## Abstract

In 1985, WHO stated that there was no justification for caesarean section (CS) rates higher than 10–15 % at population-level. While the CS rates worldwide have continued to increase in an unprecedented manner over the subsequent three decades, concern has been raised about the validity of the 1985 landmark statement. We conducted a systematic review to identify, critically appraise and synthesize the analyses of the ecologic association between CS rates and maternal, neonatal and infant outcomes. Four electronic databases were searched for ecologic studies published between 2000 and 2014 that analysed the possible association between CS rates and maternal, neonatal or infant mortality or morbidity. Two reviewers performed study selection, data extraction and quality assessment independently. We identified 11,832 unique citations and eight studies were included in the review. Seven studies correlated CS rates with maternal mortality, five with neonatal mortality, four with infant mortality, two with LBW and one with stillbirths. Except for one, all studies were cross-sectional in design and five were global analyses of national-level CS rates versus mortality outcomes. Although the overall quality of the studies was acceptable; only two studies controlled for socio-economic factors and none controlled for clinical or demographic characteristics of the population. In unadjusted analyses, authors found a strong inverse relationship between CS rates and the mortality outcomes so that maternal, neonatal and infant mortality decrease as CS rates increase up to a certain threshold. In the eight studies included in this review, this threshold was at CS rates between 9 and 16 %. However, in the two studies that adjusted for socio-economic factors, this relationship was either weakened or disappeared after controlling for these confounders. CS rates above the threshold of 9–16 % were not associated with decreases in mortality outcomes regardless of adjustments. Our findings could be interpreted to mean that at CS rates below this threshold, socio-economic development may be driving the ecologic association between CS rates and mortality. On the other hand, at rates higher than this threshold, there is no association between CS and mortality outcomes regardless of adjustment. The ecological association between CS rates and relevant morbidity outcomes needs to be evaluated before drawing more definite conclusions at population level.

**Electronic supplementary material:**

The online version of this article (doi:10.1186/s12978-015-0043-6) contains supplementary material, which is available to authorized users.

## Introduction

In 1985, a panel of experts at a meeting organized by the World Health Organization (WHO) stated that there was “no justification for any region to have a caesarean section (CS) rate higher than 10–15 %” [1]. This statement was based on the scarce evidence available at that time and on the CS rates observed in northern European countries which had one of among the lowest maternal and perinatal mortality worldwide. Although over time this figure has been regarded by the international community as the “optimal” CS rate, since then the rates of CS have escalated steadily in both developed and developing countries [2–5]. In the last 20 years, the clinical, scientific and public health communities have raised concern about the unprecedented increase in the use of CS and its consequences. The validity of the 1985 landmark statement has been questioned in light of three more decades of accumulated evidence, the large improvements in clinical obstetric care and the advances in methodologies to critically assess evidence and to issue recommendations [4, 6, 7].

The worldwide concern about this uncontrolled rise is not unjustified. Although CS is an effective procedure to prevent maternal and perinatal mortality and morbidity such as obstetric fistula from prolonged or obstructed labour or birth asphyxia, it is not without risks and it has been associated with short- and long-term complications (e.g. infection or haemorrhage in the index delivery, and uterine rupture or placentation problems in future pregnancies) [6, 8–10].

The proportion of CS at population level is a measure of the level of access to, and use of, an obstetric intervention proven to be effective in saving lives. It has served as a proxy measure for governments, policy-makers and public health professionals for assessing progress in maternal and infant health, and for monitoring emergency obstetric and resource use [11]. Determining what is the optimal CS rate at population level (i.e. the minimum and maximum rates to meet the needs for CS and at the same time avoid medically unnecessary operations) is not a trivial task. Several ecologic studies have tried to address this issue by examining the association between the mode of birth and maternal and infant outcomes at the population level [2, 12–14]. However, these analyses adopted different methodologies and provided different interpretations. To the best of our knowledge, there has been no systematic review of these studies so far. The objective of this systematic review was to identify ecological studies available in the literature that analysed the association between CS rates and maternal, neonatal or infant outcomes, assess their quality, evaluate the methodologies used, and synthesize their findings.

## Methods

We followed the reporting recommendations of the PRISMA statement [15] and the Meta-analysis Of Observational Studies in Epidemiology group (MOOSE) [16].

### Selection criteria

#### Type of study design

Studies were eligible for inclusion if they were ecologic in design, whether cross-sectional or longitudinal. Ecologic studies involve comparisons and analysis of groups, usually defined by geopolitical boundaries, rather than individuals [17].

#### Type of outcomes

Studies were eligible if they presented CS rates at population-level (e.g. regional or national) and at least one of the following outcomes: (a) Maternal outcomes: maternal mortality, hysterectomy, intra- or post-partum blood transfusion, maternal admission to ICU, prolonged maternal hospital stay or post-partum infection; (b) Newborn/infant outcomes: perinatal mortality, stillbirth, neonatal mortality, infant mortality, admission to NICU, birth asphyxia, need for mechanical ventilation, prolonged neonatal hospital stay, low-birth-weight (LBW) or preterm birth.

#### Type of population

Population-based studies regardless of socioeconomic or demographic characteristics were eligible for inclusion. A population-based study refers to a study pertaining to a general population defined by geopolitical boundaries. Reports including only women with specific demographic or obstetrical characteristics (e.g. specific maternal or gestational age, specific birthweight, or only nulliparas), or medical conditions (e.g. only HIV positive or diabetic women) were excluded. Given the improvement in practices and outcomes over time, reports providing data collected before 2000 were included only if they contained data beyond the year 2000 (e.g. a study that provided data from 1997 to 2007).

#### Search strategy and process of study identification, selection and data extraction

Four electronic databases were searched (Pubmed, Embase, LILACS and CINAHL) for studies published from January 1 2000 to March 2 2014. There were no language restrictions. The search strategy used the terms caesarean section and the outcomes listed above with synonyms and adapted to each electronic database (complete search strategy is described in Additional file 1).

The citations identified through this search strategy were processed using the EndNote® software and duplicates were excluded. The title and abstracts of unique citations were screened for potentially relevant studies. When a citation was considered relevant or the information in the title/abstract was insufficient to reach a decision, the full texts were retrieved and read. The references of all articles selected for full-text evaluation were searched for additional studies.

A specific data extraction form was created to collect the following information from each study: 1) objectives; 2) main characteristics; 3) methodology and analytical model; 4) population characteristics; 5) CS rates; and 6) health outcomes.

#### Quality assessment

There is no validated tool to assess the quality of ecologic studies. We used and adapted the checklist proposed by Dufault et al. which evaluates aspects related to study design, statistical methodology and reporting quality of ecologic studies [18]. For each study the assessment was based on 15 items with a maximum score of 21 points (Table 1); 12 points for study design, 6 for statistical methodology and 3 for quality of reporting (Additional file 2). For the analytic methodology, if the distribution of the data could not meet the assumption of the methods used, the methods were considered as inappropriately used and given a score of “0”. Otherwise, a study received a score of “1”. Assessment of the analytical methodology was determined by two authors (JZ, JFY). With regard to the flexibility of the method for model fitting and goodness-of-fit, methods that fitted the data better were given one additional point (i.e. LOWESS, piecewise regression and fractional polynomial regression). Thus, the score of the analytic methodology ranged from 0 to 2. A full description of each of the six statistical methods used in the studies included, and the assessment of the theoretical strengths and limitations of each method are presented in Additional file 3.

**Table 1:** Quality assessment criteria for ecologic studies, adapted from Dufault et al. [18]

Study design (max = 12)	
Design	Cross-sectional vs longitudinal
Sample size	Number of ecologic units included in the analysis as proportion of the total number of units, e.g. 100 countries of a total of 180 worldwide would be 55 %.
Unbiased inclusion of units	Were the units included representative of the group for which inferences are being drawn? For example, for worldwide inferences, inclusion of only developed countries would be biased
Level of data aggregation	Population to which the units refer to
Level of inference	Use of the results of the analysis of the study’s sample data to draw inferences for individuals or groups (ecologic)
Prespecification of ecologic units	Where the ecologic units selected to suit the hypothesis? (as opposed to selection motivated by convenience or necessity)
Outcomes of interest included	Inclusion of all relevant outcomes (i.e. maternal and neonatal mortality and morbidity) or only of some outcomes.
Source of data	Validity of the sources of data to represent the level that it refers to (e.g. the CS rate for one single hospital in one city would be an inadequate source of data to represent the national CS rate).
Statistical methodology (max = 6)	
Analytic methodology	All statistical methods are acceptable as long as they are used appropriately. A score was assigned based on the sophistication and flexibility of the method.
Validity of regression	Did the adjustment have at least 10 units per covariate?
Use of covariates	Did authors adjust analysis for desirable variables? Examples of socio-economic covariates: GDP or HDI. Examples of clinical covariates: proportion of women with diabetes or hypertensive disorders or obesity.
Proper adjustment for covariates	Are the outcomes standardized or adjusted for certain factors before model adjustment? For standardized or adjusted outcomes, the standardized or adjusted factors should be included in the adjustment model. If standardized/adjusted outcomes are not used, this criterion is considered to have been met.
Quality of reporting (max = 3)	
Statement of study design	Did the authors present key elements of study design in the paper?
Justification of study design	Did the authors justify the ecologic analysis, the rational and the specific objectives, including any prespecified hypotheses?
Discussion of cross-level bias and limitations	Did the authors caution readers about the limitations of the ecologic design, the ecologic fallacy, the impossibility of extrapolating to a different level?

*CS* Caesarean Section, *GDP* Gross Domestic Product, *HDI* Human Development Index

The process of screening, study selection, data extraction and quality assessment was performed in duplicate by two reviewers independently (APB, MRT) and any discrepancies were discussed until a consensus was reached.

## Results

**Fig. 1:**
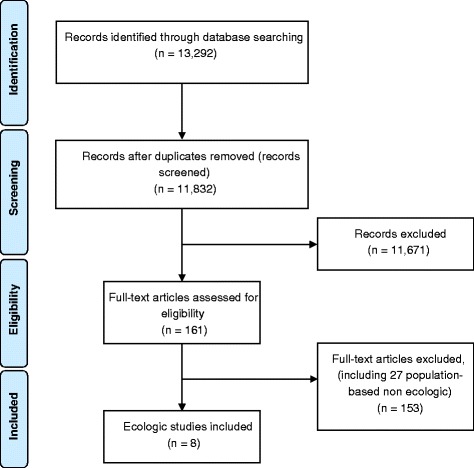
Flowchart of the systematic review: identification and selection of studies

The search strategy yielded a total of 13,292 citations which were reduced to 11,832 unique citations after the exclusion of duplicates (Fig. 1). The reviewers selected 161 citations for full-text evaluation, eight of which fulfilled the selection criteria and were included in the review [2, 4, 12–14, 19–21]. Table 2 summarizes the main characteristics of each study, range of CS, source of data, statistical methods used, results, interpretation and total quality scores. All studies were published between 2004 and 2014, and except for one [4], they were cross-sectional in design. Seven studies used data at the national level and one (from Brazil) [21] used data at the state level. Five studies [2, 12–14, 20] analysed worldwide national estimates of CS rates versus outcomes, based on the latest available data. The other three analysed a smaller set of population: one focused on 19 highly developed countries [4]; one included 18 Arab countries exclusively [19]; and one assessed state-level data from Brazil [21]. Seven studies correlated CS rates with maternal mortality, five with neonatal mortality, four with infant mortality, two with LBW and one with stillbirths (See Table 2).

**Table 2:** Main characteristics, results and interpretation of eight ecological studies included in the systematic review

Study	Period, data sets and source	Outcomes	CS range	Design, Statistical method and adjustment factors	Quality scoring (max = 21)	Results and interpretation	Considerations for socio-economic factors
Althabe et al. 2006 [12]	1991–2003	● Maternal mortality	0.4–40 %)	Cross-sectional	16	The association between CS and MMR and NMR is different among countries. In medium- and high-income countries, there is no association between CS and MMR and NMR; in low-income countries, as CS rates increase, maternal and neonatal mortality decease. An arbitrarily selected 10 % CS rate threshold seems to have particular implications: a system with <10 % CS rate would be unlikely to cover the medical needs.	When adjusting for the considered factors (socio-economic), the observed association in low-income countries became non-significant for MMR. For NMR, the association remained but weakened. No adjustment was made for clinical factors.
	119 countries grouped as low-, medium- and high-income		(median 12.9 %)	Linear regression models			
		● Neonatal mortality (early)		Adjustment for:			
	Main sources: DHS for developing countries, routine statistical surveillance systems or government reports for developed countries.			● Gross National Income			
				● Proportion of skilled birth attendant			
				● Proportion of literate population			
Betrán et al. 2007 [2]	1992–2003	● Maternal mortality	0.4–40.5 % (weighted average 15 %)	Cross-sectional	15	In countries with high mortality, CS rate has a strong inverse association with MMR, NMR and IMR. This association weakens as mortality decreases. In low mortality countries the interpretation of the association is ambiguous. Data could support the suggestion that above a certain ceiling, higher CS rates may be associated with poorer outcomes.	No adjustment was made (neither for socio-economic or clinical factors). Authors acknowledged that most likely these factors are probably important confounders and that rising CS rates possibly mirrored a change in demographic or clinical risk profile in pregnant women.
	126 countries (89 % of global live births)						
				LOWESS plots			
		● Neonatal mortality					
	Main sources: DHS for developing countries, routine statistical surveillance systems or government reports for developed countries.						
		● Infant mortality					
Jurdi et al. 2004 [19]	1995–2001	● Maternal mortality	1.4–16 %	Cross-sectional	15	In this group of 18 countries there is a strong inverse association between CS and MMR and IMR. This is a heterogeneous group of countries with very diverse socio-economic and health indicators. Only 3 countries had CS rates above 15 % (Lebanon 15.1 %, Qatar 15.9 %, and Bahrain 16 %).	No adjustment was made (neither for socio-economic or clinical factors). But authors report, a significant positive association between CS and urban population, female literacy and Gross Domestic Product per capita.
	18 Arab countries			Spearman’s rank correlation (bivariate associations)			
	Main sources: DHS or PAPCHILD surveys, UNFPA reports.	● Infant mortality					
McClure et al. 2007 [20]	Not reported	● Maternal mortality	Not available	Cross-sectional	15	In developing countries, as CS rates increased from 0 to 10–13 %, both MMR (0–10 %) and stillbirth (0–13 %) rates decreased sharply. Above 10 % CS rate, there was no significant association. In developed countries, no relationship was found.	Although this study stratifies by developed/developing countries, no further adjustment was attempted (neither for socio-economic or clinical factors).
				Piecewise regression models to explore if these relationships were consistent across the entire range of values; stepwise regression identified structural breaks in the regression lines. The sample was split at the breaks and least squares regression models were created for each of the sub-samples. Correlation and linear regression analyses were conducted.			
		● Stillbirth					
	188 countries grouped as developed (HDI > 0.80, n = 35) and developing n = 153)						
	Main sources: World Health Report 2005						
Silva et al. 2010 [21]	1995–2007 (correlation for 2005)	● Low birth weight	22–54 %^a^	Cross-sectional	15	LBW rate was not correlated with CS rate. However, data suggested a non-linear trend: up to a CS rate = 30 %, LBW rates tended to decline as CS increased. For CS rates >30 %, LBW rates tended to increase with CS. Data support the hypothesis that increasing use of medical interventions in more developed settings may increase LBW rates.	No adjustment was made (neither for socio-economic or clinical factors).
				LOWESS regression and Spearman’s rank correlation (for testing)			
	Brazil, 27 states						
	Main sources: Government database						
Volpe et al. 2011 [13]	2000–2009	● Maternal mortality	0.4–41.9 % (median 13.8 %)	Cross-sectional	15	In countries with CS rates <15 %, higher CS rates were associated with lower MMR, NMR or IMR, and lower rates of LBW. In countries with CS rates >15 %, CS were not significantly associated with IMR or MMR (for MMR and CS, a marginally significant positive correlation was found). There was no evidence that CS > 15 % correlates to poorer, nor to better, maternal or child mortality rate outcomes.	No adjustment was made (neither for socio-economic or clinical factors).
	193 countries						
		● Neonatal mortality					
	Main sources: DHS for developing countries, routine statistical surveillance systems or government reports for developed countries.	● Infant mortality		Non-linear exponential models were compared to quadratic models to regress IMR, NMR, MMR and LBWR rates to CS rate. The goodness-of-fit of models was compared using Akaike’s Information Criteria (AIC).			
		● Low birth weight					
Ye et al. 2014 [4]	1980–2010	● Maternal mortality	CS range first year: 6.2–23 %	Longitudinal analysis	18	Most of the countries have experienced sharp increases in CS rates. Once CS rate reached 10 %, with adjustment for HDI and GDP, further increases in CS rate had no impact on MMR, NMR or IMR. Country-level CS rates above 10–15 % are hardly justified from the medical perspective.	Unadjusted analysis showed decline in mortality rates with increasing CS rates (up to 15 % for MMR and 20 % for NMR and IMR). After adjustment for HDI and GDP, the relationship disappeared and the curves become flat for CS rates above 10 %. The data points for CS rates <10 % were not sufficient to draw conclusions. No adjustment was made for clinical factors.
	19 developed countries			Two-level fractional			
	Main sources: routine statistical surveillance systems or government reports.	● Neonatal mortality	CS range last year: 14.3–32.2 %	polynomial model			
				Adjustment for:			
				● Human Development Index (HDI)			
				● Gross Domestic Product (GDP)			
		● Infant mortality					
Zizza et al. 2011 [14]	1994–2008	● Maternal mortality	0.4–42.3 %	Cross-sectional	15	The analysis showed an inverse association between CS rates and MMR, and NMR for all geographical areas except for Europe. The piecewise regression provided the breakpoint beyond which an increased CS rate does not reflect an improvement in health care. The CS values for this breakpoint for NMR and MMR are 16 % and 9 %, respectively. For NMR, after 16 % there is a trend reversal; for MMR, after 9 %, it reaches a plateau.	No adjustment was made (neither for socio-economic or clinical factors).
	142 countries	● Neonatal mortality	(weighted average 14.8 %)	Analysis of covariance (Ancova) and piecewise regressions			
	Main sources: DHS for developing countries, routine statistical surveillance systems or government reports for developed countries.						

*CS* Caesarean section, *MMR* Maternal Mortality Rate, *LBW* Low birth weight, *NMR* Neonatal Mortality Rate, *IMR* Infant Mortality Rate, *DHS* Demographic and Health Surveys, *HDI* Human Development Index

^a^Estimated from graph

**Fig. 2:**
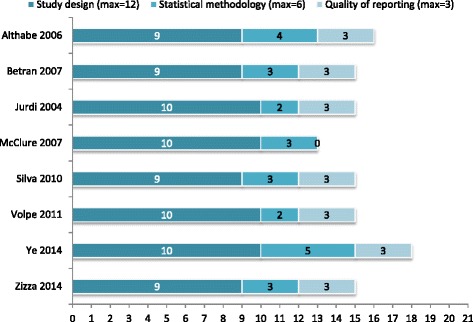
Methodological quality of the eight ecologic studies included in the review assessed under three aspects: study design, statistical methodology and quality of reporting. Maximum score, 21 points (12 for study design, 6 for statistical methodology and 3 for reporting)

The quality of the eight included studies was, in general, acceptable; all scored between 13 and 18 out of a maximum of 21 points (see Fig. 2 and Table 2). *Quality of the reporting* was the domain with the highest mean score (2 · 6 points/maximum of 3). The average score for *study design* was 9 · 5/maximum of 12 (range 9–10) and for the *statistical methodology,* it was 3 · 1/maximum of 6 (range 2–5). The most common weakness in the *study design* domain was that the outcomes included were restricted mainly to maternal, newborn and infant mortality, with the exception of three studies that included stillbirth and/or LBW [13, 20, 21]. In the statistical domain, none of the studies adjusted for clinical variables and only two studies adjusted for socio-economic variables (e.g. Gross National Income, Human Development Index) ─very likely confounders in this ecological association [4, 12]. The individual quality assessment for the eight studies is presented in Additional file 4.

A meta-analysis was not deemed appropriate given the different methodologies, country classifications and statistical methods used to assess the association. The overall results and interpretation of each study are summarized in Table 2. Five worldwide cross-sectional studies used essentially the same source of data for each country but applied different statistical models in their analyses [2, 12–14, 20]. Without controlling for any potential confounders, these five studies reported a strong and inverse association between CS rate and mortality outcomes, i.e. maternal, neonatal and infant mortality decrease as CS rates increase, up to a certain CS rate. Above that CS rate, the association no longer exists and further increases in CS rates are not associated with better outcomes. One study acknowledged that the interpretation of the association in countries with lower levels of mortality was ambiguous, and the data might even suggest that above a certain level, CS rates may be associated with an increase in adverse outcomes [2]. This hypothesis was not confirmed by another study which however used a less flexible method (an exponential regression model) for statistical analysis [13]. Among these five studies, two stratified countries by development/income level [12, 20], but only one study controlled for socio-economic development (Table 2) [12]. After adjusting, Althabe et al. reported that the observed inverse association became non-significant for maternal mortality and weakened for neonatal mortality [12].

The authors of five cross-sectional studies estimated the point at which the association between CS rates and outcomes changed [2, 12–14, 20]. This point ranged from 9 % to 16 %, and at CS rates above this threshold, there was no longer an association between increasing CS rates and reduced maternal or infant mortality. Althabe et al. established this threshold at CS rates of 10 % [12]. The unadjusted findings from Betrán et al. and Volpe et al. suggested that at CS rates higher than 15 %, there was no strong ecologic association [2, 13]. Two studies presented different thresholds according to the outcomes, both unadjusted. McClure et al. showed no significant association between increasing CS rates above 10 % and 13 % and decreasing maternal mortality and stillbirth rate, respectively [20]. Similarly, Zizza et al. concluded that CS rates above 9 % were not associated with reduction in maternal mortality, while CS rates higher than 16 % were not associated with lower neonatal mortality, but rather a mortality increase (Table 2) [14].

Unadjusted analyses by Jurdi et al. [19] on 18 Arab countries showed a strong inverse association between CS and maternal and infant mortality. Authors noted that this was a heterogeneous group of countries with very diverse socio-economic and health profiles; only 3 countries had CS rates above 15 % (Lebanon 15.1 %, Qatar 15.9 %, and Bahrain 16 %). Silva et al. [21] analysed the unadjusted correlation between CS rates and LBW in 27 Brazilian states and found no association between these two variables. However, the authors noted that while LBW rates tended to decrease as CS rates increased up to a CS rate of 30 %, above this threshold LBW rates tended to increase with increasing CS rates (Table 2).

The study by Ye and colleagues was the only one using a longitudinal design and used data from 19 developed countries [4]. As in the global cross-sectional studies, the unadjusted analysis showed a decline in mortality rates with increasing CS rates (up to 15 % for maternal mortality and 20 % for neonatal and infant mortality). After adjusting for Human Development index (HDI) and Gross Domestic Product (GDP) per capita, the relationship disappeared. While there was some indication of a marginal decrease in mortality up to 10 %, the number of data points was not sufficient to draw reliable conclusions for CS below 10 %. In this analysis, all countries included presented levels of development that would allow all women who need a CS to receive this intervention and, at the same time, some of these countries showed relatively low CS rates.

## Discussion

This review identified eight ecologic studies analysing the association between CS rates and maternal, newborn or infant outcomes. All but one used nationally representative data to assess this association. In unadjusted analyses, the threshold for the strong inverse ecologic relationship between CS rate and mortality outcomes (maternal, neonatal and infant mortality) appears to be between 9–16 %. However, in the two analyses that adjusted for socio-economic development [4, 12], the negative association between CS rate and mortality was either substantially weakened or disappeared. For CS rates over this threshold of 9–16 %, there was no association between CS rates and mortality outcomes, with or without adjustment for socio-economic development.

Confounding is one of the major threats when studying ecological associations and unfortunately, only two studies in this review controlled for potential factors or proxies. Given the lack of association in the analysis adjusted for socio-economic development, it is very likely that the inverse association found in unadjusted analysis for countries with lower CS rates may reflect a correlation between mortality and other health determinants such as access to health care, health system factors or general socio-economic conditions. Thus, the importance of Ye’s analysis lies in the longitudinal design, that may overcome some of the deficiencies of a cross-sectional analysis, and in the efforts to minimize the confounding effects of socio-economic factors by including only countries with high development/income where the necessary health services are generally accessible and thus are not a limiting factor to receive a CS. Although Ye’s analysis could not draw conclusions for CS below 10 %, this study indicates that in highly developed countries, CS rates above 10–15 % do not seem justified in terms of improved mortality. However, since Ye’s analysis included only developed countries, the question remains as to how Ye’s findings apply to other countries. A global longitudinal analysis with proper adjustment for confounding factors could address some of these issues.

The 1985 WHO recommendation focused on the rate above which further increases of CS rates might not be necessary from a medical perspective. In this respect, the findings of this review are unequivocal. The associations found in this systematic review, with or without adjustment, in essence do not contradict the 1985 recommendation. However, it is important to note that analyses for population level CS rates should not be taken as recommendations for facility level or individual provider level practice. The obstetric population case-mix, organization structure and circumstances in which each facility operates may vary dramatically from facility to facility which, in turn, can justify variations in the CS rates at each hospital. Furthermore, the current situation in some countries requires country-specific assessment. For example, in countries like Brazil where over 50 % of the births were by CS in 2010 [22], it may not be safe or advisable to achieve a CS rate of 15 % in the short term because of the large number of women with previous CS who might require a repeat CS in future pregnancies, even if programmes to encourage trial of labour for eligible women are implemented. The lack of expertise with assisted vaginal deliveries in some settings could be a major impediment to reducing CS rates. Developing and implementing appropriate training and maintaining skills in assisted vaginal deliveries is essential in order to promote the use of forceps and vacuum extractor as a safe alternative to CS in certain cases of prolonged second stage of labour.

On the other hand, an overall CS rate of 15 %, for example, does not ensure that women who require a CS for medical reasons actually receive this intervention, particularly in developing countries where equitable access and delivery of health care interventions continue to be a challenge [3]. In settings with low or very low CS rates where there are not enough skilled health professionals and/or equipment or infrastructure to ensure the safe provision of a caesarean section, caution should also be exercised when trying to increase the levels of CS.

This is the first review on the ecologic association between rates of CS and maternal or infant outcomes and provides a systematic assessment and qualitative evaluation of the primary studies, the methodologies and analyses used as well as the results and interpretations made by the different authors. Since there are no validated instruments to assess the quality of ecologic studies, we created a tool for this purpose, building upon the checklist proposed by Dufault et al. which was developed on the basis of a bibliometric review to assess the quality of modern cross-sectional ecologic studies [18]. We believe our checklist covers the most important aspects for assessment of the quality of these studies and all but one study scored 15 or more out of a maximum of 21 points. However, although all studies included in this review were rated as having acceptable quality, caution needs to be exercised given the inherent limitations of ecologic studies and in particular the insufficient adjustment for confounders in the vast majority of these studies. Concern due to lack of international guidelines for strengthening the evaluation and reporting of ecologic studies has been expressed and we hope that our checklist can contribute to the process of expanding the STROBE statement to ecologic studies [23, 24].

The availability of nationally representative data for the variables analysed in the primary studies and the source of data were acceptable. For developed countries, data came from surveillance systems or national surveys from government offices while for developing countries, the main source of data was the Demographic and Health Surveys (DHS). These sources are used for major international monitoring efforts such as the Millennium Development Goals and the United Nations Interagency Maternal Mortality Estimates [25, 26]. In addition, the rate of CS is relatively easy to collect from surveys or routine statistical information systems and its reliability has been recognized for national and global monitoring [27]. The directness and clarity of its definition also warrant higher reproducibility than other indicators such as the incidence of pre-eclampsia or post-partum haemorrhage.

The limitations of our results start by the very nature of the design of the primary studies. Ecologic associations are difficult to interpret because an association does not imply causality [17]. In addition, confounding is an important source of bias and only two of the eight studies included in this review controlled for potential confounders by using socio-economic indicators available at the national level as proxies for major determinants of health outcomes. Adjustment for other clinical or demographic characteristics at the country level (e.g. obesity, pre-eclampsia, diabetes, parity or age) would lead to better models but these are not readily available and none of the included studies controlled for them. Likewise, most of the studies analysed only mortality indicators because these are more readily available at the national level in large international databases. As mortality is a rare outcome, especially in developed countries, and CS is an intervention that can prevent only a small proportion of the maternal mortality, it would have been important to assess the association between CS rates and morbidity outcomes (e.g. infection, haemorrhage, prolonged hospital stay) or outcomes often cited as the reasons for maternal preference for CS, such as avoiding severe perineal trauma and/or pelvic floor damage. However, these are difficult to obtain at the national level and the lack of standardized definitions can be an additional source of bias.

Despite the need for global monitoring efforts to track progress at country level, monitoring CS rates at population level alone is of limited value. Additional references and tools need to be provided particularly at hospital level to achieve and maintain rates of CS that would result in the best maternal and perinatal outcomes [28–31]. In order to go beyond ecologic associations, countries with reliable health information systems should conduct analyses at national or subnational levels to appropriately assess the association between CS rates and outcomes, they should also include morbidity indicators and control for confounding factors. Studies at country level should also explore potential differences in the optimal CS rate between countries due, for example, to population differences between races.

In conclusion, this systematic review of ecologic studies found that increases in CS rates are associated with decreases in maternal, newborn and infant mortality up to CS rates of around 9–16 % but only when analyses do not control for confounders. When adjusting for socio-economic factors, the association disappears. This could be interpreted to mean that at rates below this threshold, socio-economic development rather than the CS rate may be the major determinant for mortality. On the other hand, at CS rates higher than this threshold there is no association with mortality outcomes regardless of adjustments, and increases in CS above this level do not further reduce mortality.

## Additional files

Additional file 1:
**Complete search strategy.**
Additional file 2:
**Quality assessment checklist for ecologic studies.**
Additional file 3:
**Full description of the statistical models used in the eight ecological studies, including assessment of the theoretical strengths and limitations of each method.**
Additional file 4:
**Individual quality assessment of the included studies.**
