# Supplementary material for: What is the optimal rate of caesarean section at population level? A systematic review of ecologic studies
Source: Reprod Health. 2015 Jun 21;12:57. doi: 10.1186/s12978-015-0043-6 (PMC4496821; doi:10.1186/s12978-015-0043-6)
Supplement: Additional file 1: — Complete search strategy. [file 12978_2015_43_MOESM1_ESM.docx]

| **#** | **Searches** |
| --- | --- |
| 1 | 'cesarean section'/exp OR 'cesarean section kit'/exp OR Caesarean*:ti,ab OR cesarean*:ti,ab OR ‘caesarea’:ti,ab OR fetectomy*:ti,ab OR cesarotom*:ti,ab OR caesarotom*:ti,ab OR ‘sectio caesarea’:ti,ab OR ‘abdominal delivery’:ti,ab OR ‘abdominal deliveries’:ti,ab OR ‘c section’:ti,ab OR ‘c sections’:ti,ab OR postcesarean*:ti,ab OR postcaesarean*:ti,ab |
| 2 | 'infant mortality'/exp OR ‘infant mortality’:ti,ab OR ‘infant mortalities’:ti,ab OR ‘infantile morality’:ti,ab OR ‘infant mortalities’:ti,ab OR (postneonatal OR neonatal OR maternal) NEAR/3 (mortalit* OR death*) OR (postneonatal OR neonatal OR maternal) NEAR/3 (mortalit* OR death*) OR ‘maternal mortality'/exp OR 'hysterectomy'/exp OR hysterectom*:ti,ab OR 'blood transfusion'/exp OR (blood NEAR/2 transfusion*) OR 'length of stay'/exp OR length NEAR/2 (stay OR stays) OR Hospital* NEAR/3 (stay OR stays) OR 'artificial ventilation'/exp OR (mechanical OR controlled OR artificial) NEAR/2 (respiration OR ventilation) OR 'newborn hypoxia'/exp OR ‘asphyxia neonatorum’:TI,AB OR ‘newborn hypoxia’:ti,ab OR ‘neonatal anoxia’:ti,ab OR ‘neonatal asphyxia’:TI,AB OR ‘neonatal hypoxia’:TI,AB OR ‘neonate asphyxia’:TI,AB OR ‘neonatus hypoxia’:ti,ab OR ‘newborn asphyxia’:ti,ab OR ‘premature labour’/exp OR 'puerperal infection'/exp OR 'puerperal infection':ti,ab OR ‘postpartum infection’:ti,ab OR 'puerperal infections':ti,ab OR ‘postpartum infections’:ti,ab OR 'postpartum hemorrhage'/exp OR ‘fluxus postpartum’:TI,AB OR ‘postpartum hemorrhage’:TI,AB OR ‘lochia’:TI,AB OR ‘ post partum hemorrhage’:TI,AB OR ‘postpartal hemorrhage’:TI,AB OR ‘postpartum bleeding’:TI,AB OR ‘ postpartum haemorrhage’:TI,AB OR ‘puerperal hemorrhage’:TI,AB OR ‘postpartum hemorrhage’:TI,AB OR 'intensive care'/exp |
| 3 | #1 AND #2 |
| 4 | ‘ abstract report’/exp OR ‘ conference paper’/exp OR ‘ editorial’/exp OR ‘erratum’/exp OR ‘ letter’/exp OR ‘ note’/exp OR 'case report'/exp OR 'case study'/exp OR 'arts and illustration' OR 'publication'/exp |
| 5 | #3 NOT #4 |
| 6 | #5 AND (2000:py OR 2001:py OR 2002:py OR 2003:py OR 2004:py OR 2005:py OR 2006:py OR 2007:py OR 2008:py OR 2009:py OR 2010:py OR 2011:py OR 2012:py OR 2013:py OR 2014:py) |

**Annex 1: Search strategy**

EMBASE

PUBMED

#1 "Cesarean Section"[Mesh] OR (Cesarean Section*) OR (Cesarean Section rate OR (C-section) OR ((C-section) RATE) OR (Caesarean section rate*) OR (Cesarean delivery rates) OR (Cesarean delivery) OR (Caesarean sections birth) OR (Delivery, Abdominal) OR (Abdominal Deliveries) OR (Deliveries, Abdominal) OR (Caesarean Section) OR (Caesarean Sections) OR (Abdominal Delivery) OR (C-Section (OB)) OR (C Section (OB)) OR (C-Sections (OB)) OR (Postcesarean Section)

#2 "Infant Mortality"[Mesh] OR (Infant Mortalit*) OR (Postneonatal Mortality) OR (Neonatal Mortality) OR OR ("Perinatal Mortality"[Mesh]) OR "Maternal Mortality"[Mesh] OR (Maternal Mortalit*) OR "Hysterectomy"[Mesh] OR "Blood Transfusion"[Mesh] OR "Length of Stay"[Mesh] OR (Stay Length*) OR (Hospital Stay*) OR "Respiration, Artificial"[Mesh] OR "Asphyxia Neonatorum"[Mesh] OR "Puerperal Infection"[Mesh] OR ("Postpartum Hemorrhage"[Mesh]) OR ("Intensive Care"[Mesh]) OR ("Intensive Care, Neonatal"[Mesh] )

#3 #1 AND #2

LILACS interface IAHx

#1 mh: Cesarean Section  OR Cesárea OR (Abdominal Delivery) OR (Delivery, Abdominal)  OR (Parto Abdominal)  OR (Postcesarean Section) OR (Cesarean Section$)

#2 mh:Mortalidade Infantil  OR (Mortalidade de Menores de 1 Ano de Idade) OR (Mortalidade Infantil por Risco Específico) OR (Mortalidade Infantil por Unidade Territorial) OR (Mortalidade Infantil Tardia) OR (Mortalidade do Lactente) OR (Mortalidade Neonatal) OR (Mortalidade Pós-Neonatal)  OR MH:[E05.318.308.985.550.475](javascript:void(submit_GET_METHOD('028105','028105-1','hierarchic')))$ OR MH:[L01.280.975.550.475](javascript:void(submit_GET_METHOD('028105','028105-2','hierarchic')))$ OR MH:[N01.224.935.698.489](javascript:void(submit_GET_METHOD('028105','028105-3','hierarchic')))$ OR MH:[N06.850.505.400.975.550.475](javascript:void(submit_GET_METHOD('028105','028105-4','hierarchic')))$ OR MH:[N06.850.520.308.985.550.475](javascript:void(submit_GET_METHOD('028105','028105-5','hierarchic')))$ OR MH:[SP3.076.187.173.164](javascript:void(submit_GET_METHOD('028105','028105-6','hierarchic')))$ OR MH:[SP4.011.127.413.639.905.376](javascript:void(submit_GET_METHOD('028105','028105-7','hierarchic')))$ OR MH:[SP5.006.052.168.154.110](javascript:void(submit_GET_METHOD('028105','028105-8','hierarchic')))$ OR (Infant Mortality by Specific Risk) OR (Child Mortality by Geographic Zone) OR (Late Infant Mortality) OR (Infant Mortality, Late) OR (Neonatal Mortality) OR (Post-Neonatal Mortality) OR (Postneonatal Mortality) OR MH:Mortalidade Perinatal OR (Perinatal Mortality) OR (Mortalidad Perinatal) OR MH:[E05.318.308.985.550.700](javascript:void(submit_GET_METHOD('052870','052870-1','hierarchic')))$ OR MH:[L01.280.975.550.700](javascript:void(submit_GET_METHOD('052870','052870-2','hierarchic')))$ OR MH:[N01.224.935.698.739](javascript:void(submit_GET_METHOD('052870','052870-3','hierarchic')))$ OR MH:[N06.850.505.400.975.550.700](javascript:void(submit_GET_METHOD('052870','052870-4','hierarchic')))$ OR MH:[N06.850.520.308.985.550.700](javascript:void(submit_GET_METHOD('052870','052870-5','hierarchic')))$ OR MH:[SP5.006.052.168.154.105](javascript:void(submit_GET_METHOD('052870','052870-6','hierarchic')))$ OR MH:"Hysterectomy" OR MH:"Blood Transfusion" OR MH:"Length of Stay" OR (Stay Length$) OR (Hospital Stay$) OR MH:"Respiration, Artificial" OR MH:"Asphyxia Neonatorum" OR MH:"Puerperal Infection" OR MH:"Postpartum Hemorrhage" OR MH:"Intensive Care" OR MH:"Intensive Care, Neonatal"

#3 #1 AND #2

CINHAL

#1 "Cesarean Section" OR (Cesarean Section*) OR (Cesarean Section rate OR (C-section) OR ((C-section) RATE) OR (Caesarean section rate*) OR (Cesarean delivery rates) OR (Cesarean delivery) OR (Caesarean sections birth) OR (Delivery, Abdominal) OR (Abdominal Deliveries) OR (Deliveries, Abdominal) OR (Caesarean Section) OR (Caesarean Sections) OR (Abdominal Delivery) OR (C-Section (OB)) OR (C Section (OB)) OR (C-Sections (OB)) OR (Postcesarean Section)

#2 "Infant Mortality" OR (Infant Mortalit*) OR (Postneonatal Mortality) OR (Neonatal Mortality) OR OR ("Perinatal Mortality") OR "Maternal Mortality" OR (Maternal Mortalit*) OR "Hysterectomy" OR "Blood Transfusion" OR "Length of Stay" OR (Stay Length*) OR (Hospital Stay*) OR "Respiration, Artificial" OR "Asphyxia Neonatorum" OR "Puerperal Infection" OR ("Postpartum Hemorrhage") OR ("Intensive Care") OR ("Intensive Care, Neonatal")

#3 #1 AND #2
