# Supplementary material for: What is the optimal rate of caesarean section at population level? A systematic review of ecologic studies
Source: Reprod Health. 2015 Jun 21;12:57. doi: 10.1186/s12978-015-0043-6 (PMC4496821; doi:10.1186/s12978-015-0043-6)
Supplement: Additional file 2: — Quality assessment checklist for ecologic studies. [file 12978_2015_43_MOESM2_ESM.docx]

**Annex 2: Quality assessment of ecologic studies included in the systematic review: Template and definitions**

| **Evaluation criterion** | **Categories** | **Definition** | **Points (max=21)** |
| --- | --- | --- | --- |
| **STUDY DESIGN (max=12)** | | | |
| Design | Cross-sectional  Longitudinal | If it is a multi-level design (e.g. ecologic + individual), the study is upgraded one point (e.g. cross-sectional + multi-level receives 2 points) | 1  2 |
| Sample size | < 80% units  ≥ 80% units | Number of ecologic units included in the analysis as proportion of the total number of units, e.g. 119 countries of a total of 180 worldwide would be 66%. | 0  1 |
| Unbiased inclusion of units | No  Yes | Were the units included representative of the group for which inferences are being drawn? For example, for worldwide inferences, inclusion of only developed countries would be biased. | 0  1 |
| Level of data aggregation | Other than below  Regional, State  National | Population to which the units refer to. “Other” may be: city, race groups. | 1  2  3 |
| Level of inference | Individual or unclear  Ecologic | Use of the results of the analysis of the study’s sample data to draw inferences for individuals or groups (ecologic). | 0  1 |
| Prespecification of ecologic units | No  Yes | Where the ecologic units selected to suit the hypothesis? (as opposed to selection motivated by convenience or necessity) | 0  1 |
| Outcomes of interest included | Some  All | Inclusion of all relevant outcomes (i.e. maternal and neonatal mortality and morbidity) or only of some outcomes. | 1  2 |
| Source of data | Inadequate  Adequate | Validity of the sources of data to represent the level that it refers to (e.g. the CS rate for one single hospital in one city would be an inadequate source of data to represent the national CS rate). | 0  1 |
| **STATISTICAL METHODOLOGY (max=6)** | | | |
| Analytic methodology | Spearman’s rank correlation, Linear least square regression models, Quadratic model, Exponential model, LOWESS, Fractional polynomial regression Piecewise regression, | All statistical methods are acceptable as long as they are used appropriately. We assign a score based on the sophistication and flexibility of the method.  1 = Spearman’s rank correlation, Linear least square regression models, Quadratic model, Exponential model  2 = LOWESS, Fractional polynomial regression, Piecewise regression, | 1  2 |
| Validity of regression | No  Yes | Did the adjustment have at least 10 units per covariate? | 0  1 |
| Use of covariates | None  Socio-economic  Socio-economic + clinical | Authors adjusted the analysis for desirable variables or not. Examples of socio-economic covariates: GDP or HDI. Examples of clinical covariates: proportion of women with diabetes or hypertensive disorders or BMI. | 0  1  2 |
| Proper adjustment for covariates (yes) | No  Yes | Are the outcomes standardized or adjusted for certain factors before model adjustment? For standardized or adjusted outcomes, the standardized or adjusted factors should be included in the adjustment model. If standardized/adjusted outcomes are not used, this criterion is considered to have been met. | 0  1 |
| **QUALITY OF REPORTING (max=3)** | | | |
| Statement of study design (yes) | No  Yes | Did the authors present key elements of study design in the paper? | 0  1 |
| Justification of study design (yes) | No  Yes | Did the authors justify the ecologic analysis, the rational and the specific objectives, including any prespecified hypotheses? | 0  1 |
| Discussion of cross-level bias and limitations (yes) | No  Yes | Did the authors caution readers about the limitations of the ecologic design, the ecologic fallacy, the impossibility of extrapolating to a different level? | 0  1 |
