# Supplementary material for: What is the optimal rate of caesarean section at population level? A systematic review of ecologic studies
Source: Reprod Health. 2015 Jun 21;12:57. doi: 10.1186/s12978-015-0043-6 (PMC4496821; doi:10.1186/s12978-015-0043-6)
Supplement: Additional file 3: — Full description of the statistical models used in the eight ecological studies, including assessment of the theoretical strengths and limitations of each method. [file 12978_2015_43_MOESM3_ESM.docx]

**Annex 3: Technical note on statistical methods in ecological studies**

Parametric and non-parametric statistical methods were used in the analysis of the association between caesarean section (CS) rates and adverse maternal and perinatal outcomes. These methods include locally weighted scatterplot smoothing (LOWESS), Spearman’s rank correlation, piecewise regression model, exponential model, quadratic model, and fractional polynomial model.

**a. LOWESS**

LOWESS is a non-parametric regression model known as locally weighted polynomial regression. The polynomial is fitted using weighted least squares, giving more weight to points near the point whose response is being estimated and less weight to points further away. A smooth curve through a set of data points obtained with this statistical technique is called a LOWESS curve.^1^

The most important advantage that LOWESS has over many other methods is that it does not require a specific function to fit all the data in the sample. Instead, one only has to provide a smoothing parameter value and the degree of the local polynomial. In addition, LOWESS is very flexible, making it ideal for modelling complex processes for which no theoretical models exist. The disadvantage of LOWESS is that it does not produce a regression function. This can make it difficult to transfer the results of one analysis to another [1-4].

**b. Spearman’s rank correlation**

Spearman’s rank correlation coefficient is the Pearson correlation coefficient between two ranked variables. It is a nonparametric measure of statistical dependence between two variables. It assesses how well the relationship between two variables can be described using a monotonic function [5].

The Spearman correlation is used when the probability distribution of the dependent variable is abnormal or unknown. It is appropriate for both continuous and discrete variables, including ordinal variables [5]. The Spearman correlation indicates the direction and closeness of association between the independent variable (x) and the dependent variable (y). But the coefficient is calculated based on the ranked variables. Changes in Spearman correlation coefficient indicates the closeness of the two variables rather than the quantitative relation between x and y measured by the Pearson correlation coefficient.

**c. Piecewise regression**

A piecewise linear function is a function composed of straight-line sections. If partitions are already known, a linear regression can be performed independently on these partitions. If partitions are not known, the residual sum of squares can be used to choose optimal separation points [6].

However, it is unreasonable to postulate that risk suddenly changed as a partition is crossed and the results may depend on the choice of cut-off points. When cut-off points are not chosen *a priori*, a data-driven inference may be suspected.

**d. Multiple linear regression models**

Linear regression is an approach for modelling the relationship between a scalar dependent variable y and one or more explanatory variables (x). A linear regression model assumes that the relationship between the dependent variable y*_i_* and the p-vector of x*_i_* is linear. Least squares is the simplest and most commonly used approach to fit linear regression model.

Linearity is one of the major assumptions of the standard linear regression model. To fit the linearity assumption, the predictor variables may be transformed (log scale, polynomial, etc.). Transformation of the predictor variable makes linear regression an extremely powerful method [7].

**e. Quadratic model**

Quadratic model is one of the frequently used polynomial regressions, a generalization of linear regression model. In polynomial regression models the relationship between the independent variable x and the dependent variable y is modelled as an nth order polynomial (n=2 for quadratic model). Polynomial regression has been used to describe nonlinear phenomena [8]. Although the goal of polynomial regression is to model a non-linear relationship between the independent and dependent variables, as a statistical estimation problem it is still a linear regression. Least squares is used to estimate regression coefficients.

It has been recognized that low order polynomial regression offers only a few curve shapes. It may not always fit the data well. High order polynomials fit the data better but often fit badly at the extremes of the observed x range. Although in theory, with enough polynomial terms one can approximate any smooth curve, in reality the number of terms required may be so large as to result in numerically unstable estimates. Polynomials greater than quadratic tend to produce artifactual turns in the fitted curve[8,9].

**f. Fractional polynomial regression**

Fractional polynomial regression is an extension of polynomial regression models. The power term of the independent variable *x* in a fractional polynomial regression model can be integer or non-integer values. The powers are chosen from a predefined set of values so that conventional polynomial regression models are a set of the family [10-12].

The fractional polynomial regression has much more flexibility than polynomial regression. But in the fractional polynomial model, *x* cannot be negative if fractional powers are used.

Despite the variety of the statistical methods used in previous ecological studies, most studies found a non-linear relationship between CS rates and maternal and perinatal outcomes. Fractional polynomial regression and LOWESS provided more flexible and, therefore, better fitting than the other methods.

References

1. Cleveland William S. Robust Locally Weighted Regression and Smoothing Scatterplots". *Journal of the American Statistical Association* 1979; **74** (368): 829–836.
2. Cleveland William S. LOWESS: A program for smoothing scatterplots by robust locally weighted regression. *The American Statistician 1981;* **35** (1): 54.
3. Cleveland William S, Devlin Susan J. "Locally-Weighted Regression: An Approach to Regression Analysis by Local Fitting. *Journal of the American Statistical Association* 1988; **83** (403): 596–610.
4. Wiki, the free encyclopedia. Local regression. http://en.wikipedia.org/wiki/Local_regression (accessed October 10, 2013).
5. Lehman Ann, O’Rourke Norm, Hatcher Larry, and Stepanski Edward J. *Jmp For Basic Univariate And Multivariate Statistics: A Step-by-step Guide*. Cary, NC: SAS Press. 2005; 123.
6. Vieth E. Fitting piecewise linear regression functions to biological responses. Journal of applied physiology 1989; 67 (1): 390–396.
7. Wiki, the free encyclopedia. Linear regression. http://en.wikipedia.org/wiki/Linear_regression#References. (accessed October 10, 2013).
8. Greenland S. Dose-response and trend analysis in epidemiology: alternatives to categorical analysis. Epidemiology 1995; 6: 356-365.
9. Wiki, the free encyclopedia. Polynomial regression. http://en.wikipedia.org/wiki/Polynomial_regression (accessed October 10, 2013).
10. Royston P, Altman DG. Regression using fractional polynomials of continuous covariates: parsimonious parametric modeling (with discussion). *Appl Stat* 1994; **43**: 429–467.
11. Royston P, Ambler G, Sarerbrei W. The use of fractional polynomials to model continuous risk variables in epidemiology.International Journal of Epidemiology 1999; 28: 964–974.
12. Sarerbrei W, Royston P. Building multivariable prognostic and diagnostic models: transformation of the predictors using fractional polynomials. *J R Stat Soc, Ser A* 1999; **162**: 71–94.
