# Supplementary material for: What is the optimal rate of caesarean section at population level? A systematic review of ecologic studies
Source: Reprod Health. 2015 Jun 21;12:57. doi: 10.1186/s12978-015-0043-6 (PMC4496821; doi:10.1186/s12978-015-0043-6)
Supplement: Additional file 4: — Individual quality assessment of the included studies. [file 12978_2015_43_MOESM4_ESM.docx]

**Annex 4: Quality assessment of eight ecologic studies included in the systematic review**

Study: Althabe 2006

| **Evaluation criterion** | **Categories** | **Points** |
| --- | --- | --- |
| **STUDY DESIGN** | | |
| Design | Cross-sectional  Longitudinal | 1 |
| Sample size | < 80% units  ≥ 80% units | 0 |
| Unbiased inclusion of units | No  Yes | 1 |
| Level of data aggregation | Other than below  Regional, State  National | 3 |
| Level of inference | Individual or unclear  Ecologic | 1 |
| Prespecification of ecologic units | No  Yes | 1 |
| Outcomes of interest included | Some  All | 1 |
| Source of data | Inadequate  Adequate | 1 |
| **STATISTICAL METHODOLOGY** | | |
| Analytic methodology | Spearman’s rank correlation, Linear regression model, Quadratic model, Exponential model, LOWESS, Fractional polynomial regression, Piecewise regression | 1 |
| Validity of regression | No  Yes | 1 |
| Use of covariates | None  Socio-economic  Socio-economic + clinical | 1 |
| Proper adjustment for covariates | No  Yes | 1 |
| **QUALITY OF REPORTING** | | |
| Statement of study design | No  Yes | 1 |
| Justification of study design | No  Yes | 1 |
| Discussion of cross-level bias and limitations | No  Yes | 1 |
| **TOTAL POINTS 16** | | |

Study: Betrán 2007

| **Evaluation criterion** | **Categories** | **Points** |
| --- | --- | --- |
| **STUDY DESIGN** | | |
| Design | Cross-sectional  Longitudinal | 1 |
| Sample size | < 80% units  ≥ 80% units | 0 |
| Unbiased inclusion of units | No  Yes | 1 |
| Level of data aggregation | Other than below  Regional, State  National | 3 |
| Level of inference | Individual or unclear  Ecologic | 1 |
| Prespecification of ecologic units | No  Yes | 1 |
| Outcomes of interest included | Some  All | 1 |
| Source of data | Inadequate  Adequate | 1 |
| **STATISTICAL METHODOLOGY** | | |
| Analytic methodology | Spearman’s rank correlation, Linear regression model, Quadratic model, Exponential model, LOWESS, Fractional polynomial regression, Piecewise regression | 2 |
| Validity of regression | No  Yes | 1 |
| Use of covariates | None  Socio-economic  Socio-economic + clinical | 0 |
| Proper adjustment for covariates | No  Yes | 0 |
| **QUALITY OF REPORTING** | | |
| Statement of study design | No  Yes | 1 |
| Justification of study design | No  Yes | 1 |
| Discussion of cross-level bias and limitations | No  Yes | 1 |
| **TOTAL POINTS 15** | | |

Study: Jurdi 2004

| **Evaluation criterion** | **Categories** | **Points** |
| --- | --- | --- |
| **STUDY DESIGN** | | |
| Design | Cross-sectional  Longitudinal | 1 |
| Sample size | < 80% units  ≥ 80% units | 1 |
| Unbiased inclusion of units | No  Yes | 1 |
| Level of data aggregation | Other than below  Regional, State  National | 3 |
| Level of inference | Individual or unclear  Ecologic | 1 |
| Prespecification of ecologic units | No  Yes | 1 |
| Outcomes of interest included | Some  All | 1 |
| Source of data | Inadequate  Adequate | 1 |
| **STATISTICAL METHODOLOGY** | | |
| Analytic methodology | Spearman’s rank correlation, Linear regression model, Quadratic model, Exponential model, LOWESS, Fractional polynomial regression, Piecewise regression | 1 |
| Validity of regression | No  Yes | 1 |
| Use of covariates | None  Socio-economic  Socio-economic + clinical | 0 |
| Proper adjustment for covariates | No  Yes | 0 |
| **QUALITY OF REPORTING** | | |
| Statement of study design | No  Yes | 1 |
| Justification of study design | No  Yes | 1 |
| Discussion of cross-level bias and limitations | No  Yes | 1 |
| **TOTAL POINTS 15** | | |

Study: McClure 2007

| **Evaluation criterion** | **Categories** | **Points** |
| --- | --- | --- |
| **STUDY DESIGN** | | |
| Design | Cross-sectional  Longitudinal | 1 |
| Sample size | < 80% units  ≥ 80% units | 1 |
| Unbiased inclusion of units | No  Yes | 1 |
| Level of data aggregation | Other than below  Regional, State  National | 3 |
| Level of inference | Individual or unclear  Ecologic | 1 |
| Prespecification of ecologic units | No  Yes | 1 |
| Outcomes of interest included | Some  All | 1 |
| Source of data | Inadequate  Adequate | 1 |
| **STATISTICAL METHODOLOGY** | | |
| Analytic methodology | Spearman’s rank correlation, Linear regression model, Quadratic model, Exponential model, LOWESS, Fractional polynomial regression, Piecewise regression | 2 |
| Validity of regression | No  Yes | 1 |
| Use of covariates | None  Socio-economic  Socio-economic + clinical | 0 |
| Proper adjustment for covariates | No  Yes | 0 |
| **QUALITY OF REPORTING** | | |
| Statement of study design | No  Yes | 0 |
| Justification of study design | No  Yes | 0 |
| Discussion of cross-level bias and limitations | No  Yes | 0 |
| **TOTAL POINTS 13** | | |

Study: Silva 2010

| **Evaluation criterion** | **Categories** | **Points** |
| --- | --- | --- |
| **STUDY DESIGN** | | |
| Design | Cross-sectional  Longitudinal | 1 |
| Sample size | < 80% units  ≥ 80% units | 1 |
| Unbiased inclusion of units | No  Yes | 1 |
| Level of data aggregation | Other than below  Regional, State  National | 2 |
| Level of inference | Individual or unclear  Ecologic | 1 |
| Prespecification of ecologic units | No  Yes | 1 |
| Outcomes of interest included | Some  All | 1 |
| Source of data | Inadequate  Adequate | 1 |
| **STATISTICAL METHODOLOGY** | | |
| Analytic methodology | Spearman’s rank correlation, Linear regression model, Quadratic model, Exponential model, LOWESS, Fractional polynomial regression, Piecewise regression | 2 |
| Validity of regression | No  Yes | 1 |
| Use of covariates | None  Socio-economic  Socio-economic + clinical | 0 |
| Proper adjustment for covariates | No  Yes | 0 |
| **QUALITY OF REPORTING** | | |
| Statement of study design | No  Yes | 1 |
| Justification of study design | No  Yes | 1 |
| Discussion of cross-level bias and limitations | No  Yes | 1 |
| **TOTAL POINTS 15** | | |

Study: Volpe 2011

| **Evaluation criterion** | **Categories** | **Points** |
| --- | --- | --- |
| **STUDY DESIGN** | | |
| Design | Cross-sectional  Longitudinal | 1 |
| Sample size | < 80% units  ≥ 80% units | 1 |
| Unbiased inclusion of units | No  Yes | 1 |
| Level of data aggregation | Other than below  Regional, State  National | 3 |
| Level of inference | Individual or unclear  Ecologic | 1 |
| Prespecification of ecologic units | No  Yes | 1 |
| Outcomes of interest included | Some  All | 1 |
| Source of data | Inadequate  Adequate | 1 |
| **STATISTICAL METHODOLOGY** | | |
| Analytic methodology | Spearman’s rank correlation, Linear regression model, Quadratic model, Exponential model, LOWESS, Fractional polynomial regression, Piecewise regression | 1 |
| Validity of regression | No  Yes | 1 |
| Use of covariates | None  Socio-economic  Socio-economic + clinical | 0 |
| Proper adjustment for covariates | No  Yes | 0 |
| **QUALITY OF REPORTING** | | |
| Statement of study design | No  Yes | 1 |
| Justification of study design | No  Yes | 1 |
| Discussion of cross-level bias and limitations | No  Yes | 1 |
| **TOTAL POINTS 15** | | |

Study: Ye 2014

| **Evaluation criterion** | **Categories** | **Points** |
| --- | --- | --- |
| **STUDY DESIGN** | | |
| Design | Cross-sectional  Longitudinal | 2 |
| Sample size | < 80% units  ≥ 80% units | 0 |
| Unbiased inclusion of units | No  Yes | 1 |
| Level of data aggregation | Other than below  Regional, State  National | 3 |
| Level of inference | Individual or unclear  Ecologic | 1 |
| Prespecification of ecologic units | No  Yes | 1 |
| Outcomes of interest included | Some  All | 1 |
| Source of data | Inadequate  Adequate | 1 |
| **STATISTICAL METHODOLOGY** | | |
| Analytic methodology | Spearman’s rank correlation, Linear regression model, Quadratic model, Exponential model, LOWESS, Fractional polynomial regression Piecewise regression | 2 |
| Validity of regression | No  Yes | 1 |
| Use of covariates | None  Socio-economic  Socio-economic + clinical | 1 |
| Proper adjustment for covariates | No  Yes | 1 |
| **QUALITY OF REPORTING** | | |
| Statement of study design | No  Yes | 1 |
| Justification of study design | No  Yes | 1 |
| Discussion of cross-level bias and limitations | No  Yes | 1 |
| **TOTAL POINTS 18** | | |

Study: Zizza 2014

| **Evaluation criterion** | **Categories** | **Points** |
| --- | --- | --- |
| **STUDY DESIGN** | | |
| Design | Cross-sectional  Longitudinal | 1 |
| Sample size | < 80% units  ≥ 80% units | 0 |
| Unbiased inclusion of units | No  Yes | 1 |
| Level of data aggregation | Other than below  Regional, State  National | 3 |
| Level of inference | Individual or unclear  Ecologic | 1 |
| Prespecification of ecologic units | No  Yes | 1 |
| Outcomes of interest included | Some  All | 1 |
| Source of data | Inadequate  Adequate | 1 |
| **STATISTICAL METHODOLOGY** | | |
| Analytic methodology | Spearman’s rank correlation, Linear regression model, Quadratic model, Exponential model, LOWESS, Fractional polynomial regression, Piecewise regression | 2 |
| Validity of regression | No  Yes | 1 |
| Use of covariates | None  Socio-economic  Socio-economic + clinical | 0 |
| Proper adjustment for covariates | No  Yes | 0 |
| **QUALITY OF REPORTING** | | |
| Statement of study design | No  Yes | 1 |
| Justification of study design | No  Yes | 1 |
| Discussion of cross-level bias and limitations | No  Yes | 1 |
| **TOTAL POINTS 15** | | |
